# Comprehensive analysis of differences in N6-methyladenosine RNA methylomes in *Helicobacter pylori* infection

**DOI:** 10.3389/fcell.2023.1136096

**Published:** 2023-06-07

**Authors:** Huan Li, Jiahui Lin, Sha Cheng, Jingshu Chi, Ju Luo, Yu Tang, Wenfang Zhao, Yufeng Shu, Xiaoming Liu, Canxia Xu

**Affiliations:** ^1^ Department of Gastroenterology, The Third Xiangya Hospital of Central South University, Changsha, Hunan, China; ^2^ Hunan Key Laboratory of Non-Resolving Inflammation and Cancer, Central South University, Changsha, Hunan, China

**Keywords:** gastritis, *Helicobacter pylori*, MeRIP-seq, M6A, N6-methyladenosine

## Abstract

**Background:** *Helicobacter pylori* (*H.pylori*) infection is an important factor in the occurrence of human gastric diseases, but its pathogenic mechanism is not clear. N6-methyladenosine (m6A) is the most prevalent reversible methylation modification in mammalian RNA and it plays a crucial role in controlling many biological processes. However, there are no studies reported that whether *H. pylori* infection impacts the m6A methylation of stomach. In this study, we measured the overall level changes of m6A methylation of RNA under *H. pylori* infection through *in vitro* and *in vivo* experiment.

**Methods:** The total quantity of m6A was quantified in gastric tissues of clinical patients and C57 mice with *H. pylori* infection, as well as acute infection model [*H. pylori* and GES-1 cells were cocultured for 48 h at a multiplicity of infection (MOI) from of 10:1 to 50:1]. Furthermore, we performed m6A methylation sequencing and RNA-sequencing on the cell model and RNA-sequencing on animal model.

**Results:** Quantitative detection of RNA methylation showed that *H. pylori* infection group had higher m6A modification level. M6A methylation sequencing identified 2,107 significantly changed m6A methylation peaks, including 1,565 upregulated peaks and 542 downregulated peaks. A total of 2,487 mRNA was upregulated and 1,029 mRNA was downregulated. According to the comprehensive analysis of MeRIP-seq and RNA-seq, we identified 200 hypermethylation and upregulation, 129 hypermethylation but downregulation, 19 hypomethylation and downregulation and 106 hypomethylation but upregulation genes. The GO and KEGG pathway analysis of these differential methylation and regulatory genes revealed a wide range of biological functions. Moreover, combining with mice RNA-seq results, qRT- PCR showed that m6A regulators, METTL3, WTAP, FTO and ALKBH5, has significant difference; Two key genes, PTPN14 and ADAMTS1, had significant difference by qRT- PCR.

**Conclusion:** These findings provide a basis for further investigation of the role of m6A methylation modification in *H. pylori*-associated gastritis.

## Introduction

*Helicobacter pylori* (*H. pylori*), a Gram-negative microaerobic bacterium, is closely related to diseases such as gastritis, peptic ulcer and chronic gastritis (Cover and Blaser, 2009; Asano et al., 2016). It can initiate gastric carcinogenesis following the Correa cascade (Correa and Piazuelo, 2012). Once atrophy and intestinal metaplasia occur, there is still a lack of effective therapy to reverse the pathological changes, and some patients still progress to gastric cancer. Therefore, it is of great clinical significance to further explore the molecular mechanism of gastric diseases caused by *H. pylori* infection and find new intervention strategies and targets.

N6-Methyladenosine (m6A), involving methylation at the N6 position of RNA adenine, is the most prevalent RNA modification in eukaryotes (Meyer and Jaffrey, 2014; Huang et al., 2020a). In the 1970s, a study reported that there has m6A modification in mRNA and non-coding RNA of eucaryon (Desrosiers et al., 1974).

M6A is the most prevalent post-transcriptional modification of mRNAs and non-coding RNAs, which determines RNA fate, such as splicing, localization, stabilization, translation efficiency and nuclear export (Guo et al., 2021; Li et al., 2022a; Zhang et al., 2022). Recent years, more and more studies have reported that m6A plays different role during the growth and development of mammals, including embryonic development, circadian rhythm, neurogenesis, stress responses, sex determination and tumorigenesis (Pan et al., 2018; Chokkalla et al., 2020; Jiang et al., 2021; Xiao et al., 2022a). M6A modification mainly involves three enzymes, a family methyltransferase enzymes (writers), which including Methyltransferase Like 3 (METTL3), Methyltransferase Like 14 (METTL14), WT1 Associated Protein (WTAP) and et all, catalyze addition of m6A (Jiang et al., 2021) (Sun et al., 2022) (Sacco et al., 2022). The demethylase enzymes (erasers) that catalyze removal of m6A, such as alpha-ketoglutarate-dependent dioxygenase AlkB homolog 5 (ALKBH5) and fat mass and obesity-associated protein (FTO) (Roignant and Soller, 2017; Jiang et al., 2021). The m6A reader proteins can recognize the m6A-modified RNAs, which are divided into different protein families, such as IGF2 mRNA binding proteins (IGF2BP1/2/3) families, eukaryotic initiation factor (eIF) 3, the proteins contain the YT521-B homology (YTH) domain (YTHDF1/2/3 and YTHDC1/2) and et all (Jiang et al., 2021) (Shi et al., 2018) (Zhou et al., 2022). It is now clear that this reversible post-transcriptional modification is essential for gene regulation.

At present, research on stomach-related diseases m6A is mainly in gastric carcinoma and rarely in non-cancer disease. The role of m6A RNA modifications in diseases associated with *H. pylori* infection has not been investigated. In this study, we used high-throughput sequencing (MeRIP-seq) to identify the potential m6A modification of inflammation in gastric epithelial cells (GES-1) treated with *H. pylori*. Differential methylation genes (DMG), differential expression genes (DEG) and differential methylation and expression genes (DMEG) were analyzed by gene Ontology (GO) and Kyoto Encyclopedia of Genes and Genomes (KEGG) pathways to reveal the biological significance of genomes. In addition, combining with mice RNA-seq data, we used qRT- PCR tests to observe the expression of five common m6A regulatory and the three key gene, which were consistent in the sequencing results of cell model and animal model. These findings may provide new insights into the molecular mechanisms involved in *H. pylori* infection.

## Materials and methods

### Bacterial strains and cell lines

*H. pylori* was isolated from the gastric mucosa of gastric ulcer patient during gastroscopy as described (Xia et al., 2020). It was cultured in Columbia agar containing 10% sheep blood (Nanjing bianzhen Biological Technology Co., LTD., China) and antibiotics (5 mg/L cefsulodin, 5 mg/L amphotericin B, 5 mg/L trimethoprim, 10 mg/L vancomycin) (Oxoid, United Kingdom) at 37°C under microaerophilic conditions (5% O_2_, 10% CO_2_, and 85% N_2_) for 3–5 days. When the value of OD600 was 1, the bacterial concentration was approximately 2 × 10^8^ CFU/mL.

GES-1 cells were obtained from Hybribio Biotech Ltd. (Guangdong, China). The GES-1 cells were cultured in RPMI-1640 medium (Gibco, United States), containing 10% fetal bovine serum (Biological Industries, Israel) and maintained at 37°C in humidified 5% CO_2_ incubator.

### Clinical specimens

Four *H. pylori*-positive and four *H. pylori*-negative gastric tissues were collected from patients who underwent gastric biopsies at the Xiangya Third Hospital, Central South University (Changsha, China). The diagnoses were based on clinical and histological laboratory examination. All patients had signed informed consent for the study. The clinical information of patients was shown in Supplementary Table S1. This study was approved by the Ethics Committees of the Xiangya Third Hospital, Central South University.

### *H. pylori* -infected animal model

Four to five weeks old male C57 BL/6 (18–22 g) were used. All the experimental animals were foster in the Department of Laboratory Animal Science of Central South University and were housed in an experimental animal room, which meets the specific pathogen-free (SPF)-class Meets the SPF standard, to ensure an environment with 12 h of light and 12 h of darkness. The eight mice were divided into two groups: control group (*n* = 4) and *H. pylori* infection group (*n* = 4). The mice were orally gavaged with 0.3 mL *H. pylori* suspension in phosphate buffered saline (PBS) (1 × 10^9^) once daily for 9 days (repeat three times with 1 day off for three consecutive days) according to our previous study (Xia et al., 2020). The mice were only gavaged with sterile PBS in control group. The mice were sacrificed by cervical dislocation under CO_2_ narcosis at 2 weeks after last gavage. Rapid urease test (RUT) and Giemsa staining were used to verify whether mice were infected with *H. pylori* (Supplementary Figure S1).

### Cell infection model

GES-1 cells were seeded in 6-well plates until the density reached 60%–80% (∼3 × 10^5^) without *H. pylori* intervention and the cell culture medium containing no antibiotics. *H. pylori* was collected and re-suspended into antibiotic-free cell culture medium. The concentration of *H. pylori* suspension was adjusted to 1× 10^9^/mL. Then, *H. pylori* suspension was added to GES-1 cells at a MOI of 10:1–50:1 and incubated for 48 h.

### RNA extraction and qRT-PCR

Total RNA in tissues was extracted by the TRIzol reagent (Invitrogen, United States). Moreover, the extracted total RNA dissolved in RNase/DNase-free water. The ReverTra Ace qPCR RT Master Mix with gDNA Remover (Vazyme Biotech Co., Ltd., China) was used to reverse transcribe RNA in accordance with the manual. Primers for qRT-PCR were listed in Supplementary Table S2.

### Quantification of the m6A modification

Total RNA was isolated as above. The quality of RNA was analyzed using a NanoDrop1000 (Thermo Fisher, United States). The EpiQuik m6A Methylation Quantification Kit (Epigentek, P-9005-96, United States) was used to measure the global m6A enrichment of mRNA. 200 ng RNA was coated in assay wells from each sample. The m6A levels are colorimetrical quantified at a wavelength of 450 nm absorbance.

### RNA-seq and m6A-RNA immunoprecipitation sequencing

Total RNA was isolated from GES-1 cells and gastric tissue of mouse by TRIzol reagent as above. The Poly (A) RNA was purified from 50 µg total RNA using Dynabeads Oligo (dT) (Thermo Fisher, Carlsbad CA, United States) and two rounds of purification were used. Next, a Magnesium RNA Fragmentation Module was used to fragment the captured mRNA at 86°C for 7 min. Cleaved RNA fragments were incubated with m6A-specific antibody (Synaptic Systems GmbH, Goettingen, Germany) for 2 h at 4°C in IP buffer which was consist of 750 mM NaCl, 50 mM Tris-HCl and 0.5% Igepal CA-630. After performing IP, the IP product was synthesized into cDNA using reverse tran-scriptase (Invitrogen SuperScript™ II Reverse Transcriptase, CA, United States). *Escherichia coli* DNA polymerase I (NEB, United States), RNase H (NEB, United States), and dUTP Solution (Thermo Fisher, United States) which assisted the synthesis of the double-stranded DNA and the ends of the double-stranded DNA were repaired to form blunt ends. The two strands were digested with the enzyme UDG (NEB, United States) after adding an A base to both blunt ends and using magnetic beads to screen and purify the fragments according to size. Through PCR experiment, a library with a fragment size of 300 ± 50 bp was established (Supplementary Table S2). Finally, an Illumina NovaSeq™ 6000 (LC- Bio Technology Co., Ltd., Hangzhou, China) was used to sequencing with PE150 (2 bp × 150 bp paired-end) sequencing mode.

### Bioinformatics analysis

Fastp (https://github.com/OpenGene/fastp) was used for quality control on the original data and acquire clean data. HISAT2 package (http://daehwankim lab.github.io/hisat2) was used to compare the acquired clean data to the genome (human genome, version: hg19; and *mus musculus* genome, version: GRCm38). The R package exome-Peak (https://bioconductor.org/packages/exome Peak) was used to perform peak calling analysis and peak analysis of genetic difference. The IGV software (http://www.igv.org) visualized the results. HOMER (http://homer.ucsd.edu/homer/motif) and MEME2 (http://meme-suite.org) were used for motif analysis. StringTie (https://ccb.jhu.edu/softw are/stringtie) was used to determine the expression levels of all mRNAs in the input libraries. The different expression of mRNAs was selected according to thresholds of a *p*-value < 0.05 and a |log2 (fold change)| >1 with the R package edgeR (https://bioconduct or.org/packages/edgeR).

### Statistical analysis

SPSS 22.0 and GraphPad Prism 7.0 were used for data processing. The *t*-test and χ2 test were used to analyze the differences among different samples. A *p*-value less than 0.05 was considered to indicate statistical significance (*p < 0.05, **p < 0.01, ***p < 0.001, ****p < 0.0001).

## Results

### Establishment of *H. pylori* infection model *in vivo* and *in vitro*

**FIGURE 1:**
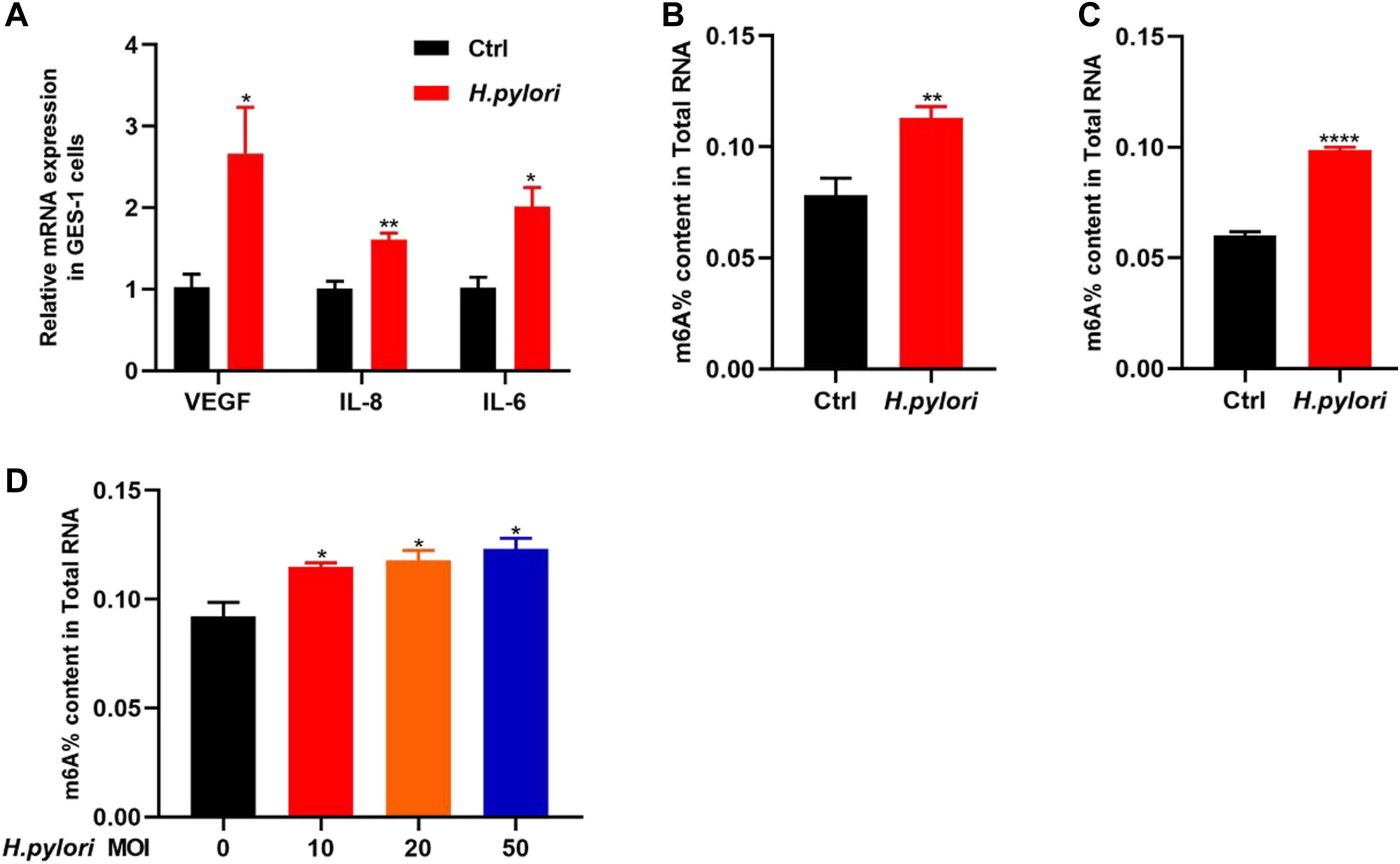
Establishment of *H. pylori* infection model and determination of total m6A **(A)** The RNA-level expression of inflammatory factors IL-8, IL-6, and VEGF after *H. pylori* infection of GES-1 cell. **(B)** The total m6A content in *H. pylori* negative and positive patients (*n* = 4). **(C)** The total m6A content in *H. pylori* negative and positive animal (*n* = 4). **(D)** The total m6A content in *H. pylori*-uninfected and *H. pylori*-infected cells in different MOI (*n* = 3). *p < 0.05; **p < 0.01; ***p < 0.001; ****p < 0.0001.

In this study, GES-1 cells were treated with *H. pylori* with a MOI of 10:1 for 48 h. We detected the mRNA expression of VEGF, IL-6 and IL-8 by qRT-PCR (Figure 1A). The results show that the expression levels of these proinflammatory factors were significantly increased in the GES-1 cells treated with *H. pylori* (*p* < 0.05). Next, we observed significant increase in the overall level of m6A methylation in *H. pylori*-infected patients and mice (Figures 1B, C), and mild significant increase in *H. pylori*-infected cells (Figure 1D).

### Overview of methylation RNA immunoprecipitation sequencing

In the MeRIP-seq library, the two sets of samples obtained an average of 41,015,657 and 44,650,725 valid reads, while in the RNA-seq library the two groups obtained an average of 37,346,096 and 40,732,363 valid reads (Supplementary Table S3). Among the IP samples, the average matching rate of valid reads in the control group and *H. pylori* group was 97.2% and 97.1%, respectively. The mean matching rates for valid reads in the input samples were 97.5% and 97.7% (Supplementary Table S4). Clean reads that can be matched to the reference genome are defined as exons, introns and intergenic sequences according to the regional information of the reference genome. The mean rates of IP and exons in the input samples were 97.39% and 97.44% for the control group and 96.1% and 96.86% for the *H. pylori*-infected group, respectively (Supplementary Figure S2).

### Profile of the m6A modification in GES-1 cells treated with *H. pylori*

**FIGURE 2:**
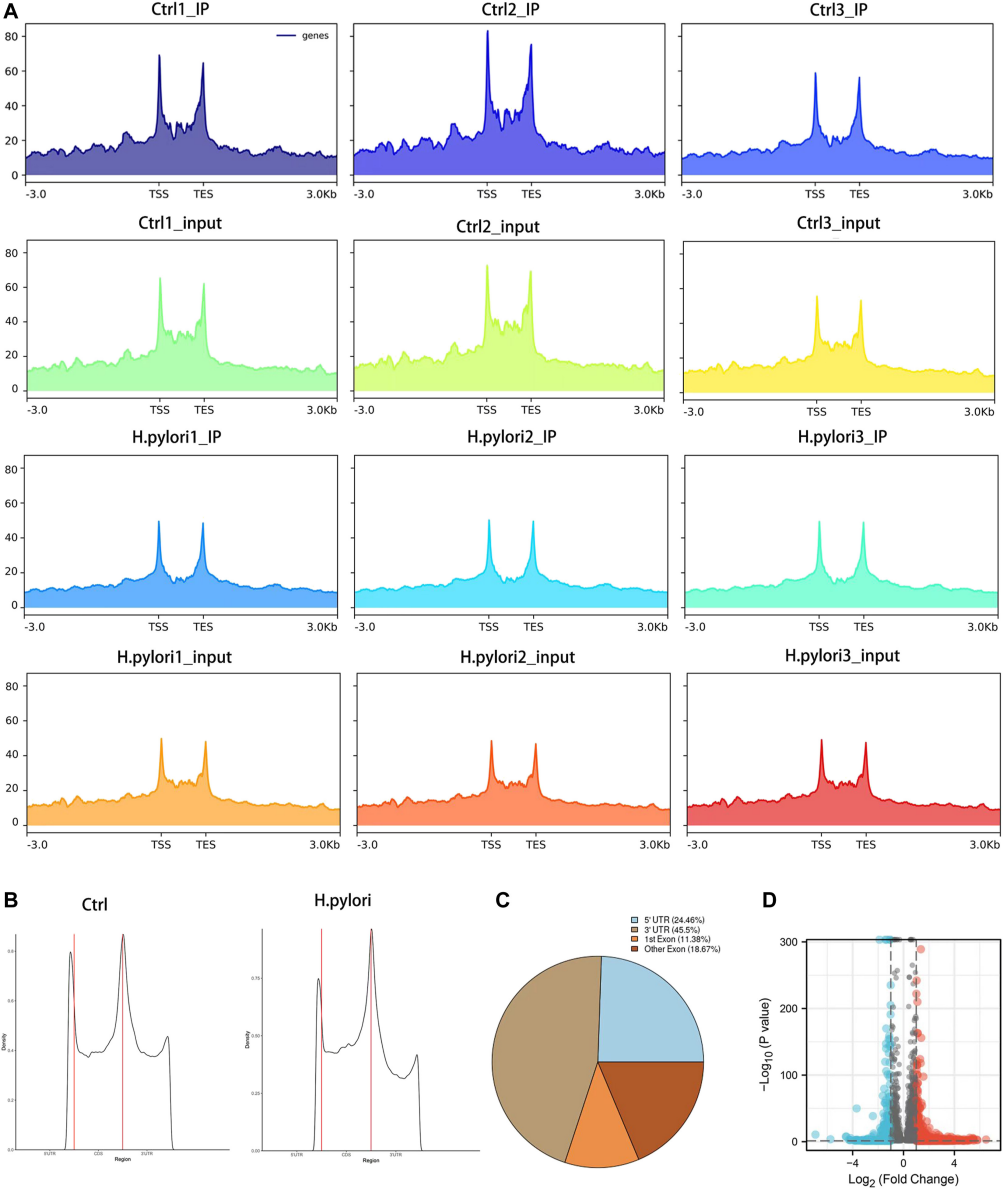
Significant dysregulation of the m6A peak during *H. pylori* infection of gastric epithelial cells. **(A)** Enrichment of peaks near the gene transcription start site. **(B,C)** Distribution of differentially methylated m6A peaks in control and *H. pylori* groups **(D)** Volcano plot of genes with differential m6A peaks (|log2(FC)|>1 and *p*-value < 0.05).

To obtain a map of m6A modifications in gastric epithelial cells infected with *H. pylori*, we used meRIP-seq to performed a transcriptome analysis of m6A modification. Combining all the peak reads, we found that the enrichment of reads was located near the transcription start site (TSS) and the transcription end site (TES) (Figure 2A). To further understand the distribution of the differential peak on the functional elements of the gene, we divided it into three regions: the 5′ untranslated region (5′ UTR), the first exon, the other exons and the 3′ UTR (Figure 2B). Meanwhile, we analyzed the distribution pattern of differential m6A methylation peaks. A total of 24.46% of the m6A methylation peaks were contained in the 5′UTR, 45.5% were enriched in the 3′ UTR, while 11.38% were enriched in the exons (Figure 2C). Under the screening conditions of |log2 (fold change)| > 1 and *p*-value < 0.05, a total of 9,097 peaks were identified in both groups. The results showed 2,107 significantly different peaks compared to the control group, of which 1,565 peaks were upregulated and 542 peaks were downregulated (Figure 2D). The top 20 distinct m6A methylation peaks are shown in Supplementary Table S5.

### Differential m6A modification is involved in important biological pathways

**FIGURE 3:**
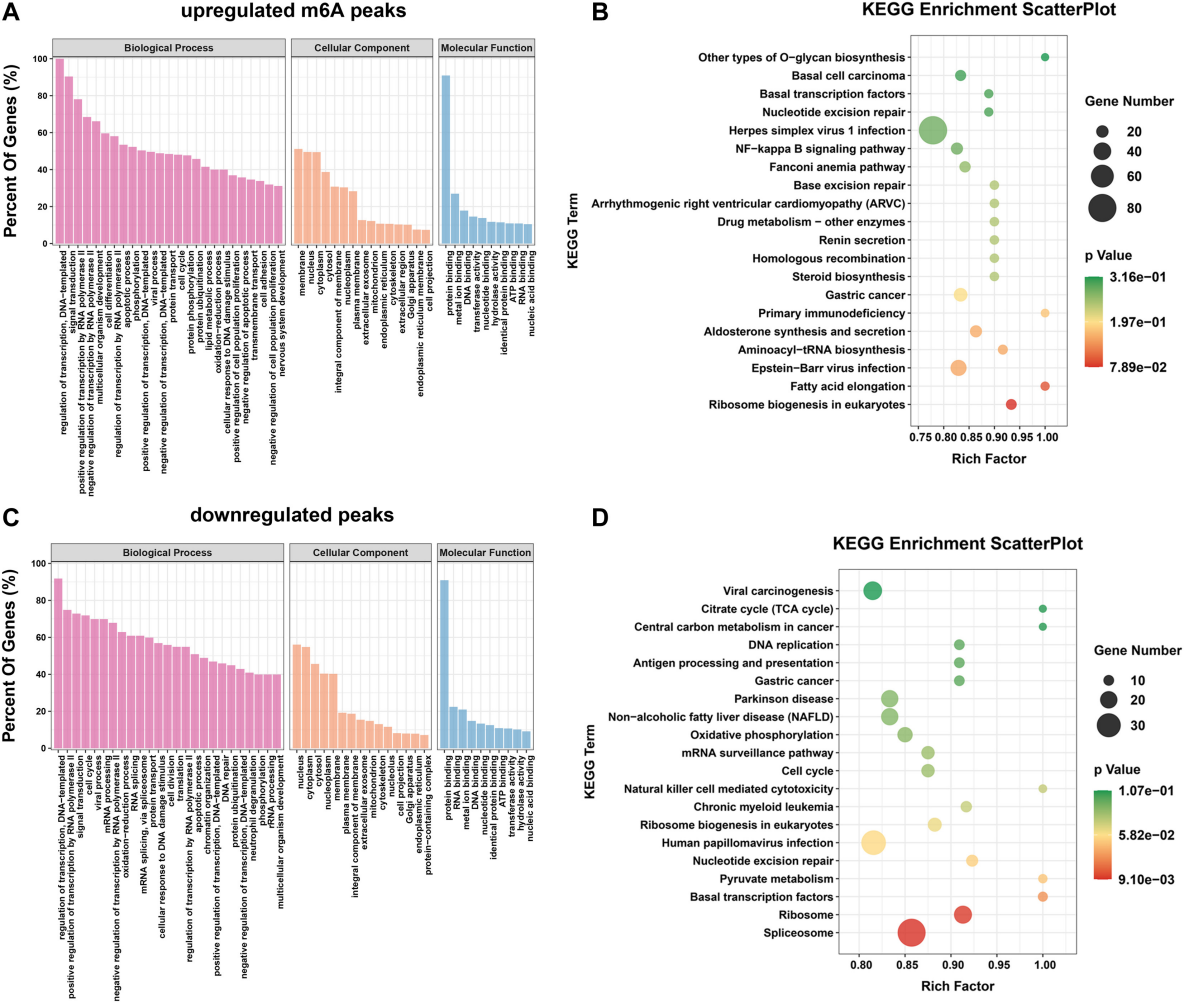
Differential m6A modifications are involved in important biological pathways. **(A,B)** GO enrichment and KEGG pathway analysis of the hypermethylated peaks. **(C,D)** GO enrichment and KEGG pathway analysis of the hypomethylated peaks.

To explore the important functions of m6A modification in *H. pylori*-induced gastric epithelial cells, GO and KEGG enrichment analyses were performed for the above m6A differential peak (DMG) genes. The GO results were classified into three categories: cellular component (CC) and biological process (BP) and molecular function (MF) categories. It can be observed that both hypermethylated and hypomethylated genes are associated with “regulation of transcription, DNA template,” “signal transduction,” “apoptotic process,” " regulation of transcription by RNA polymerase II,” “cell cycle” and “RNA splicing” (ontology: biological processes); “nucleus,” " membrane,” “cytoplasm” and “cytoplasm” (ontology: cellular components); and “protein binding,” “RNA binding,” “metal ion binding” (ontology: molecular function) (Figures 3A, C). In addition, the results of the KEGG signaling pathway analysis showed that the genes upregulated by the m6A peak were mainly enriched in “fatty acid elongation,” “primary immunodeficiency,” “Epstein-Barr virus infection,” “drug metabolism-other enzymes,” “NF-κB signaling pathway,” and “basic transcription factors” (Figure 3B); The genes downregulated by the m6A peak were mainly concentrated in “natural killer cell mediated cytotoxicity,” “basal transcription factors,” “cell cycle,” “mRNA surveillance pathway,” and “pyruvate metabolism” (Figure 3D).

### Analysis of RNA-seq differential expression genes

**FIGURE 4:**
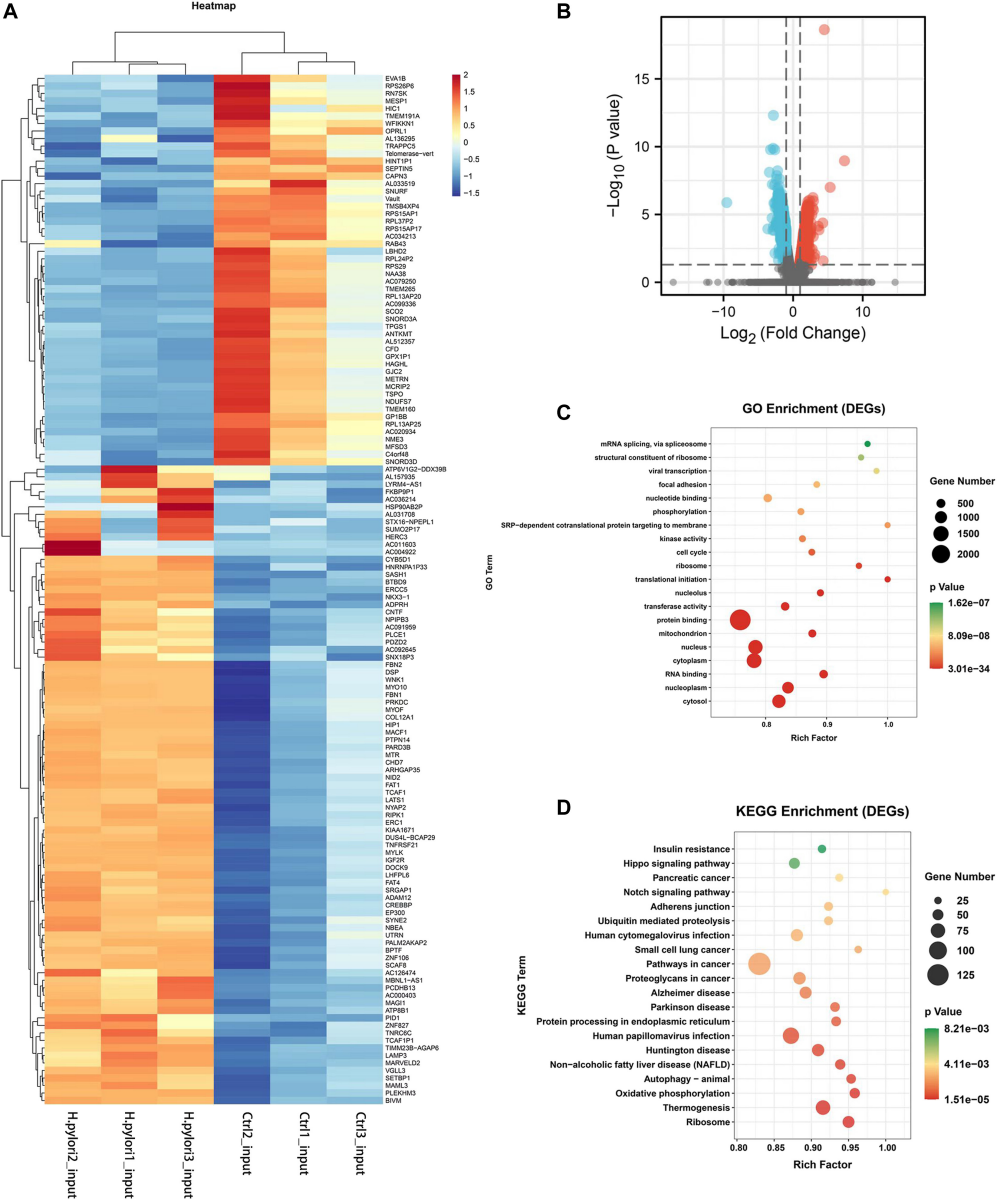
Differentially expressed gene analysis by RNA-Seq **(A)** Heat map showing differentially expressed mRNAs in three *H. pylori* input samples and three control input samples. **(B)** Volcano plot showing the differentially expressed mRNAs between *H. pylori* and control groups with statistical significance (fold change ≥ 2.0 and *p* < 0.05). **(C)** GO enrichment analysis of differential genes. **(D)** KEGG pathway analysis of differential genes.

To explore the potential relationship between m6A modifications and gene expression, differential expression gene analysis was performed using input sequencing data. By hierarchical clustering of RNA-seq data, we detected significantly different expression between the control and *H. pylori* groups (Figure 4A). We then screened the RNA-seq database for a total of 3,516 differential genes (|log2(FC)|>1 and *p*-value < 0.05) compared to control samples. Among them, 2,487 upregulated genes and 1,029 downregulated genes were identified (Figure 4B). These differential expression genes were then used for GO enrichment and KEGG pathway analysis. GO enrichment results showed these genes were significantly related to “translation initiation,” “SRP-dependent cotranslation protein targeting membranes,” “viral transcription” and “mRNA splicing, via spliceosomes” (Figure 4C). KEGG analysis showed that these genes were mainly enriched in “Notch signaling pathway,” “adherens junctions,” “Hippo signaling pathway,” “protein processing in endoplasmic reticulum,” “ubiquitin mediated proteolysis” and “oxidative phosphorylation” (Figure 4D).

### Combined analysis between m6A-seq and RNA-seq

**FIGURE 5:**
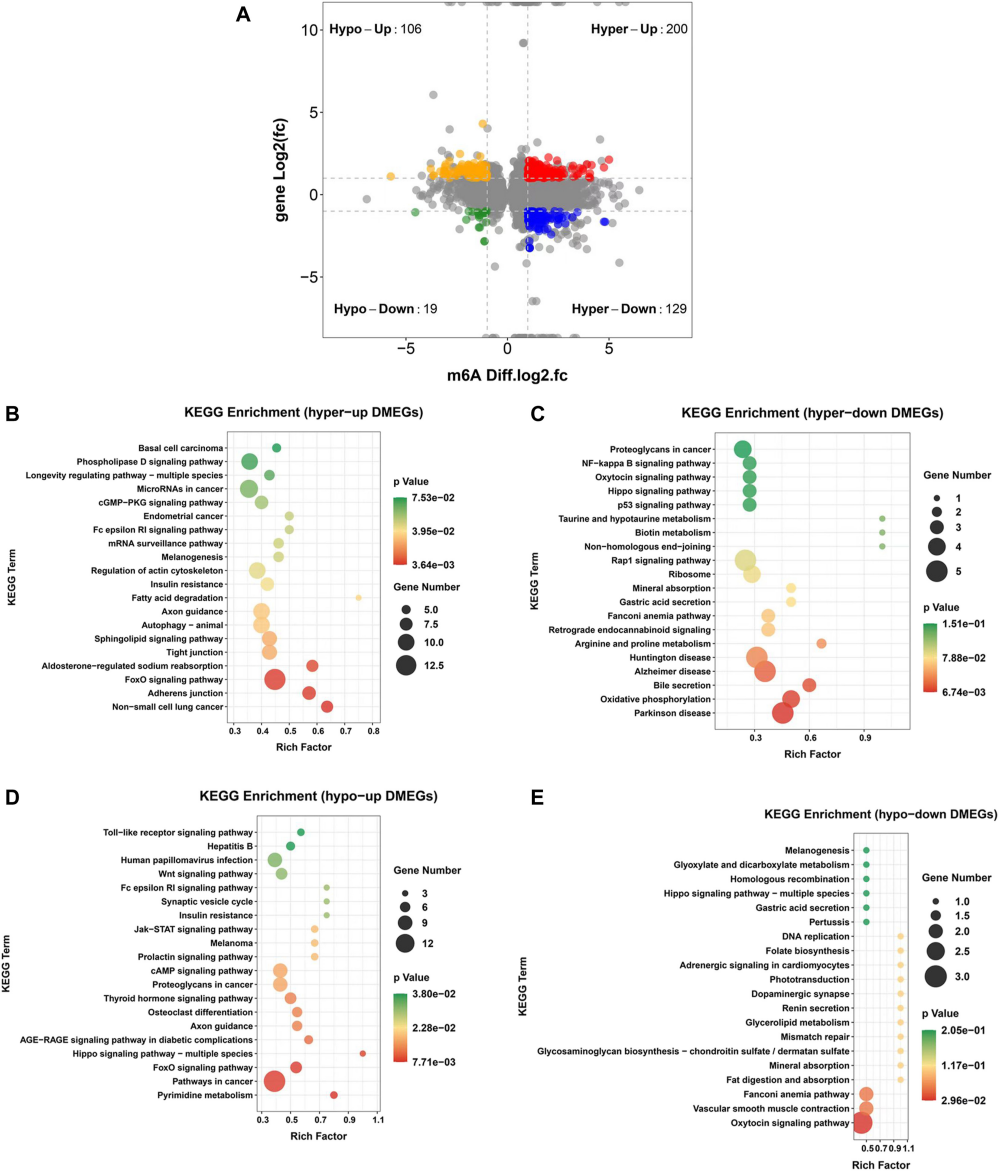
Combined analysis between m6A-Seq and RNA-Seq **(A)** Four-quadrant diagram of hyper-up, hyper-down, hypo-up, and hypo-down DMEG. **(B)** KEGG pathway analysis of hyper-up DMEG **(C)** KEGG pathway analysis of hyper-down DMEG **(D)** KEGG pathway analysis of hypo-up DMEG **(E)** KEGG pathway analysis of hypo-down DMEG.

To further explore the functional significance of m6A modifications in *H. pylori*-infected gastric epithelial cells, we investigated whether m6A methylation underlies the observed differences in expression. For this purpose, DMGs and DEGs were detected using m6A-seq data and RNA-seq data. Thereafter, a combination of m6A-seq and RNA-seq analysis classified all genes into four major groups: including 200 hypermethylated and upregulated (hypo-up), 129 hypermethylated but downregulated (hypo-down), 19 hypermethylated and downregulated (hypo-down) and 106 hypomethylated but upregulated genes or transcripts (hypo-up) (Figure 5A). Four groups of DMEG were further investigated by KEGG analysis. The results showed that hyper-up genes were mainly enriched in “Adherens junctions,” “FoxO signaling pathway” and “Fatty acid degradation” pathways (Figure 5B); in contrast, hyper-down genes were mainly enriched in “Bile secretion,” “Gastric acid secretion,” “Oxidative phosphorylation” and “NF-κappa B signaling pathway” (Figure 5C). In addition, hypo-up genes were mainly enriched in “Toll-like receptor signaling pathway,” “Wnt signaling pathway,” “Jak-STAT signaling pathway,” “cAMP signaling pathway,” “pathway in cancer” and “Hippo signaling pathway-multi-species” (Figure 5D), while hypo-down genes were mainly enriched in “Vascular smooth muscle contraction” and “DNA replication” (Figure 5E). Moreover, we list the top 20 transcripts of differential m6A modification and mRNA expression between control group and *H. pylori* group based on diff.log2.fc (Table 1).

**TABLE 1:** Top 20 transcripts of differential m6A modification and mRNA expression between control group and *H. pylori* group.

Gene name	Change	Seqnames	m6A modification change						mRNA expression change	
			Peak start	Peak end	Width	Peak region	logFC	*p*-value	logFC	*p*-value
PTPN14	Hyper-up	chr1	214,532,254	214,532,432	179	Exon	5	4.57E-02	2.13	1.75E-06
FRY	Hyper-up	chr13	32,298,645	32,298,945	301	Exon	4.75	1.74E-04	1.65	9.74E-04
TRANK1	Hyper-up	chr3	36,857,290	36,857,679	390	Exon	4.14	9.55E-04	1.79	4.63E-03
HSPA12A	Hyper-up	chr10	116,827,654	116,827,984	331	3′ UTR	4.07	1.70E-02	1.02	4.62E-02
ADAM10	Hyper-up	chr15	58,748,838	58,749,018	181	3′ UTR	4.06	5.75E-03	1.29	1.96E-02
BOLA2	Hyper-down	chr16	29,454,886	29,455,005	120	5′ UTR	4.81	3.72E-03	−1.65	5.48E-06
AL136038	Hyper-down	chr14	63,642,540	63,642,600	61	Exon	4.75	4.07E-02	−1.64	3.21E-04
QPCTL	Hyper-down	chr19	45,703,571	45,703,690	120	3′ UTR	3.44	1.74E-03	−1.07	2.95E-04
PIDD1	Hyper-down	chr11	804,682	805,011	330	5′ UTR	3.20	3.24E-03	−1.38	9.14E-05
RPS9	Hyper-down	chr19	54,224,672	54,224,881	210	Exon	2.91	1.17E-02	−1.28	7.32E-05
NSF	Hypo-up	chr17	46,640,090	46,643,106	3,017	3′ UTR	−5.75	2.34E-04	1.10	2.92E-02
ADAMTS1	Hypo-up	chr21	26,843,766	26,844,363	598	5′ UTR	−3.78	2.00E-03	1.57	2.25E-03
MAPRE2	Hypo-up	chr18	34,977,018	34,978,391	1,374	5′ UTR	−3.65	8.91E-03	1.15	1.58E-02
CEP78	Hypo-up	chr9	78,276,633	78,276,783	151	3′ UTR	−3.63	4.37E-03	1.17	1.91E-02
LAMA3	Hypo-up	chr18	23,899,378	23,899,528	151	Exon	−3.31	1.17E-02	1.42	4.32E-03
SLX1B	Hypo- down	chr16	29,457,636	29,458,189	554	3′ UTR	−4.54	1.95E-08	−1.07	2.50E-04
RPS3AP47	Hypo- down	chr15	43,115,761	43,115,907	147	Exon	−2.03	2.88E-03	−1.52	5.21E-05
RPL29P11	Hypo- down	chr3	37,016,898	37,017,014	117	Exon	−1.91	8.71E-06	−1.01	5.54E-04
WTIP	Hypo- down	chr19	34,504,119	34,504,179	61	3′ UTR	−1.73	4.47E-02	−1.04	1.35E-03
PPP1R12C	Hypo- down	chr19	55,092,432	55,093,073	642	Exon	−1.47	5.01E-13	−1.06	2.37E-04

### PPI network and hub genes were identified in DMEG

**FIGURE 6:**
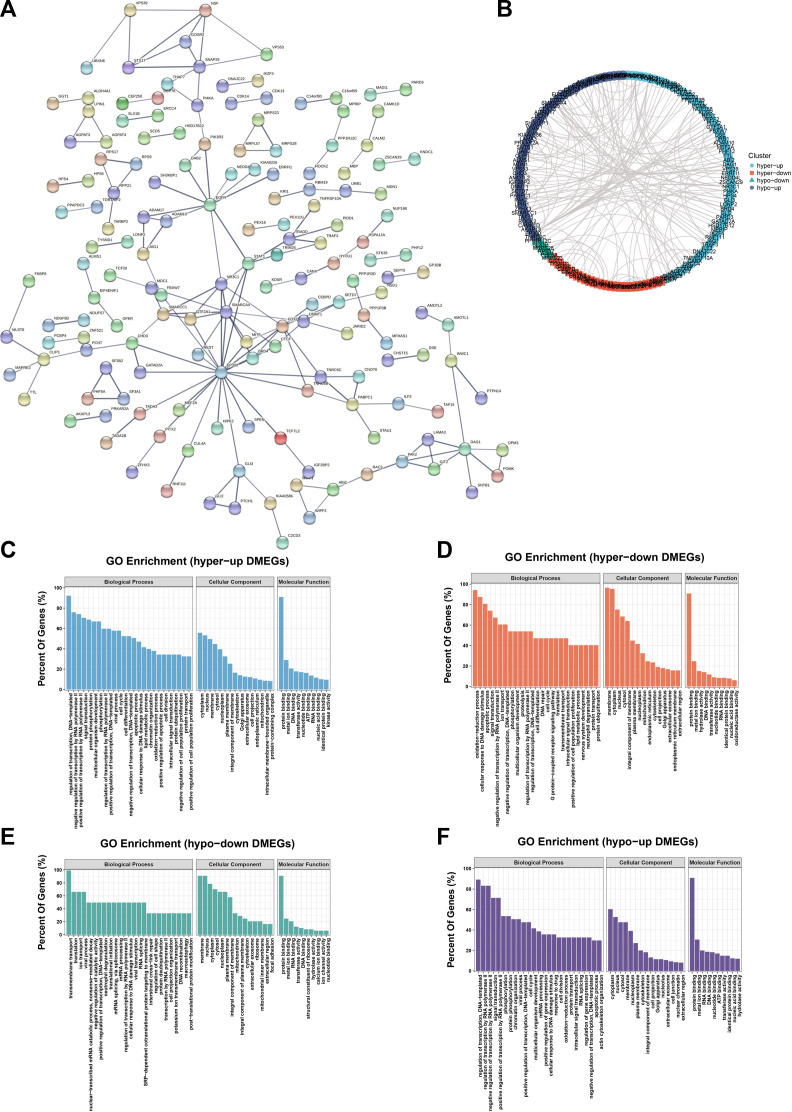
PPI networks and hub genes were found in DMEG. **(A)** PPI network of DMEG constructed from STRING database **(B)** Cytoscape was performed divided into four clusters. Blue represents hyper-up genes, orange represents hyper-down genes, green represents hypo-down genes, and purple represents hypo-up genes. **(C)** GO enrichment analysis of the hyper-up cluster in this DMEG. **(D)** GO enrichment analysis of hyper-down clusters in this DMEG. **(E)** GO enrichment analysis of the hypo-down cluster in this DMEG. **(F)** GO enrichment analysis of the hypo-up cluster in this DMEG.

The PPI network of DMEG is carried out by the STRING database (Figure 6A) and Cytoscape. As above, the network was divided into four clusters, which respectively are hyper-up, hyper-down, hypo-down and hypo-up DMEGs (Figure 6B). GO enrichment analysis was performed for each DMEG cluster to elucidate its biological functions (Figures 6C–F). PPI network interaction data are listed in Supplementary Table S6.

### Validation of differential expression genes

**FIGURE 7:**
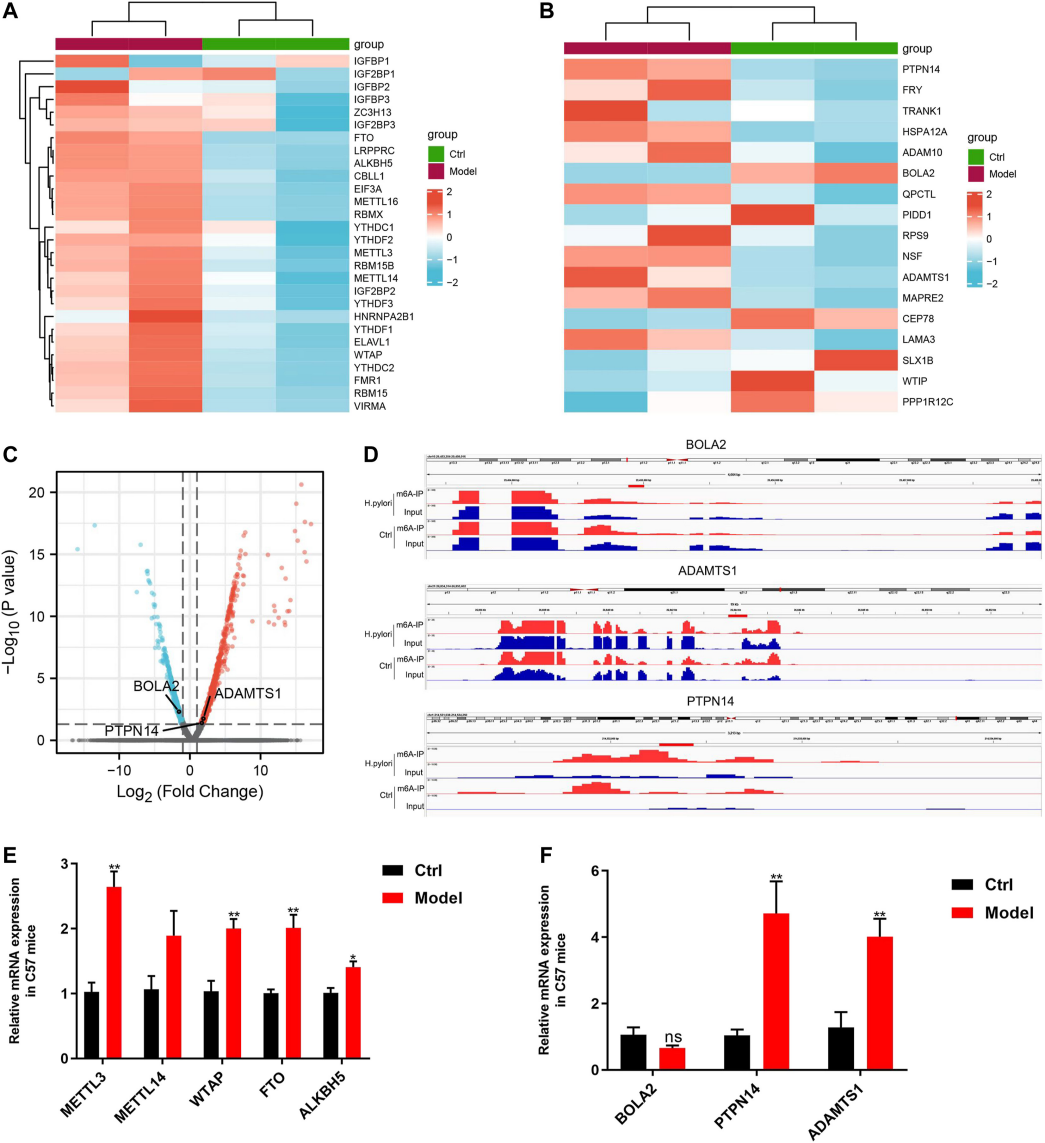
Validation of differential expression genes **(A)** Heatmap of m6A regulators in sequencing of mice samples. **(B)** RNA-seq data of mice to validate the top 20 genes from cell sequencing by Heatmap. **(C)** Volcanic map of differentially expressed genes sequencing of mice samples. **(D)** IGV visualization show the three key m6A-modified genes. **(E)** qRT- PCR results for five common m6A regulators. **(F)** qRT- PCR results of the three key m6A-modified genes.

In the RNA-seq data of mice, we analyzed the mRNA levels of 28 m6A regulators, except IGF2BP1, 27 of 28 m6A regulators showed an increasing tendency (Figure 7A). Furthermore, qRT-PCR was used to detect the expression levels of five common regulators, including METTL3, METTL14, WTAP, FTO and ALKBH5; The change trends in those genes revealed by qRT- PCR were consistent with the with the RNA-seq results, those gene all did arrive significant difference except METTL14 (Figure 7E).

We used sequencing of mice samples results to validate the expression of top 20 genes of cell sequencing (Table 1), which shows that only 3 genes were consistent, including PTPN14, BOLA2 and ADAMTS1 (Figures 7B, C). We performed IGV visualization for the three genes and all found significantly different m6A levels (Figure 7D). Moreover, except BOLA2, PTPN14 and ADAMTS1 had significant difference by qRT- PCR (Figure 7F).

## Discussion

*H. pylori* infection can damage the stomach mucosa, leading to the development of various stomach diseases which involve many pathophysiological changes. Abnormalities in m6A modifying enzymes can cause a series of diseases (Jiang et al., 2021) (Zhang et al., 2019). However, the mechanism of m6A modification in *H. pylori*-induced gastric epithelial infection remains unclear. In this study, the relationship between m6A modification profile and *H. pylori*-induced gastric epithelial infection was analyzed for the first time.

In the beginning, we found that *H. pylori* infection increase the level of m6A modification *in vitro* and *in vivo*. After that, we obtained an overview of m6A modification in *H. pylori* infection associated gastritis through MeRIP-seq. The total peak numbers of m6A revealed significant differences in m6A modification between the control and *H. pylori* groups. Therefore, we assume that m6A modification may be related to *H. pylori* -induced gastritis.

As is known to all, m6A modification of mRNA often affects the occurrence and development of the disease. In this study, we identified 2,107 significantly different peaks compared to the control, of which 1,565 peaks were upregulated and 542 peaks were downregulated. From this, we found that *H. pylori* can alter the methylation peak of GES-1. Therefore, we hypothesized that m6A modification may be associated with *H. pylori*-induced gastric epithelial infection. M6A peaks were mostly enriched near the 3′ UTR region, and these sites were m6A specific and consistent with previous studies (Huang et al., 2020a). The 3′ UTR regulates mRNA stability, localization, expression and translation of mRNA. Multiple RNA-binding proteins bind in this region to perform regulatory functions and regulate the interaction between proteins (Mayr, 2019). In addition, in *H. pylori*-infected gastric epithelial cells, differential methylation peaks were significantly enriched in “transcriptional regulation,” “RNA splicing,” “signal transduction,” “apoptotic processes” and “cell cycle.” Previous studies indicated that *H. pylori* involve the regulation of apoptosis, proliferation and the cell cycle (Hirata et al., 2001; Nozawa et al., 2002; Ding et al., 2008). This suggests a conserved and fundamental role of m6A in the regulation of development and cell fate specification. The hypermethylation peaks were mainly concentrated in “fatty acid elongation,” “EBV infection,” “drug metabolism,” “NF-κB signaling pathway” “EBV infection,” “drug metabolism,” “NF-κB signaling pathway” and “basic transcription factor” pathways. NF-κB is a key regulator of the immune response against *H. pylori* infection and is known to modulate genes involved in the control of inflammation, cell proliferation and apoptosis (Lamb and Chen, 2010; Chaturvedi et al., 2011; Shu et al., 2022). The hypomethylation peaks were mainly enriched in “natural killer cell-mediated cytotoxicity,” “basic transcription factors,” “cell cycle,” “mRNA surveillance pathway,” “basic transcription factors.” The mRNA surveillance pathway” and “pyruvate metabolism” pathways. This evidence indicates that m6A modification is probably associated with *H. pylori* -associated gastritis.

In order to clarify the mechanism of m6A affecting the process of *H. pylori* infection in gastric epithelial cells, we combined m6A methylation group with transcription group to find the key signaling pathways affected by m6A modification. Previous studies have shown that during the time course of *H. pylori* infection, *H. pylori* infection destroys the integrity of the gastric mucosa. *H. pylori* induces classical and alternative NF-κB signaling pathways through its effector ADP-L-glycero-β-D-manno-heptose (ADP-heptose), leading to deleterious gastric pathophysiology (Maubach et al., 2022). It has also been shown that *H. pylori* can induce a signaling cascade by activating the Toll-like receptor pathway, which ultimately leads to the transcription of pro-and anti-inflammatory cytokines and type I interferons (Peek et al., 2010). The Hippo signaling pathway appears to be a protective pathway in the host-pathogen conflict that generates an inflammatory environment, cellular injury, and epithelial renewal and differentiation, limiting the loss of gastric epithelial properties prior to adenocarcinoma development, which may be beneficial for *H. pylori* colonization and chronic infection (Molina-Castro et al., 2020). As in previous studies, some classical pathways regarding *H. pylori* causing gastric disease were significantly enriched in the present study. These include NF-κB signaling pathway (Keates et al., 1997; Sasaran et al., 2021), p53 signaling pathway (Cai et al., 2021; Imai et al., 2021), Hippo signaling pathway, Toll-like receptor signaling pathway (Lam et al., 2022), and Wnt signaling pathway (Abdi et al., 2021). This evidence also suggests that m6A modification may be associated with *H. pylori*-induced gastric disease.

In the past few years, numerous studies have illustrated the biological effects of m6A modification on RNA. On the one hand, the m6A methylation process is reversible, and this mark on RNA can be written or erased under various stimuli and biological factors (Feng et al., 2022; Liu et al., 2022; Wang et al., 2022). On the other hand, m6A can affect RNA processing and metabolism through a variety of mechanisms, including selective polyadenylation, selective splicing, RNA stability, RNA export, RNA degradation, and translation (Wang et al., 2014; Zhao et al., 2014; Coots et al., 2017; Hong et al., 2022). Thus, m6A up- or downregulates gene expression in a complex and context-dependent manner. For this reason, we observed four groups of DMEGs in the present study, which are hyper-up, hyper-down, hypo-up, and hypo-down. Our functional enrichment analysis showed that these four groups of DMEGs are associated with essential and different biological processes. Many previous studies reported that m6A modification is involved in different biological processes, such as transcriptional regulation, signal transduction, and the DNA damage response (Jia et al., 2011; Zheng et al., 2013; Hong et al., 2022). Our results are corresponded to previously these published studies.

In the RNA-seq data of mice, we found numerous m6A regulators were found to have changes. Except IGF2BP1, other m6A regulators showed an increasing tendency. To further verify the results of sequencing, we observed the expression of five common regulators by qRT- PCR which showed similar results to sequencing. Those genes (METTL3, WTAP, FTO and ALKBH5) all did arrive significant difference except METTL14. METTL3, as one of the core components of the m6A methyltransferase complex, has been found to be closely related to multiple signaling pathways, such as the JAK/STAT (Yao et al., 2019), MAPK/NF-κB (Li et al., 2020a), PI3K/AKT (Bi et al., 2021), and Wnt/β-catenin pathways (Cui et al., 2020). WTAP has been reported to be associated with a number of signaling pathways, such as TGFβ (Li et al., 2020b), hippo (Hu et al., 2020), NF-κB (Li et al., 2021), and Hedgehog pathways (Wei et al., 2022). FTO is associated with various signaling pathways, for example, PKA/CREB (Hu et al., 2022), TNF-α (Li et al., 2022b), ERK (Xiao et al., 2022b), WNT (Kim et al., 2022) and JAK2/STAT3 pathways (Shen et al., 2021). ALKBH5 is involved in many signaling pathways, including WNT (Lin et al., 2022), PTEN/AKT (He et al., 2021), NF-κB (Qu et al., 2022), AKT (Wang et al., 2020). Interestingly, many studies have shown that *H. pylori* infection is closely related to these signaling pathways. *H. pylori* can active the expression of STAT1 and PD-L1 which may prevent immune surveillance in the gastric mucosa, allowing premalignant lesions to progress to gastric cancer (Li et al., 2022c). *H. pylori* can induce injuries to the stomach through MAPK/NF-κB pathway (Shu et al., 2022). *H. pylori* can induce the occurrence of gastric carcinogenesis at the early stage by activating the PI3K/Akt signaling pathway (Xu et al., 2018). *H. pylori* infection activated WNT/β-catenin signaling pathway by upregulating to induce gastritis (Zuo et al., 2022). Judging from these, m6A regulators may also involve in regulating different signaling pathways in *H. pylori*-associated gastritis.

Moreover, the results of sequencing of mice samples were used to validate the expression of top 20 genes of cell sequencing, which found three genes, PTPN14, BOLA2 and ADAMTS1, that are consistent. Furthermore, qRT- PCR showed PTPN14 and ADAMTS1 had significant difference. Although there was no significant difference in BOLA2 expression, there was a downward trend. *H. pylori* was able to significantly upregulate PTPN14 and ADAMTS1 mRNA expression levels. At present, there are no studies relationship between these three genes and *H. pylori*-infected diseases. Many studies showed that PTPN14 has different function, such as suppressing the occurrence and development of tumor (Hatterschide et al., 2022), blunting the formation of atherosclerosis (Yang et al., 2021a) and promoting inflammation and fibrosis (Fu et al., 2020; Lin et al., 2021). Previous studies confirmed that ADAMTS1 is involved in inhibiting the proliferation, polarization and migration of tumor (Li et al., 2015; de Assis Lima et al., 2021), affecting the quality of oocytes and embryonic development potential (Yang et al., 2021b) and promoting collagen production (Toba et al., 2016). In addition, a recent study revealed that YTHDF2 inhibited ADAMTS1 expression and promoted sperm adhesion through m6A/mRNA pathway (Huang et al., 2020b). Above all, these results suggest that m6A is likely to exhibit an as-yet- unknown function in the process by *H. pylori* -induced gastritis.

In summary, we can infer that m6A methylation was shown to play a role in *H. pylori*-induced gastritis. Though the mechanism of m6A-regulated gastritis is not clearly understood, we provide the first m6A transcriptome profile of gastritis and an initial map revealing the function of m6A modification in gastritis using advanced technologies, thereby contributing critical insights for further research on the role of m6A in gastritis. These findings provide a basis for further investigation of the role of m6A methylation modification in *H. pylori* infection of the gastric mucosa. However, the finding into clinical scenario may be limited by the lack of verification of the expression and distribution of m6A related regulatory molecules in clinical samples of *H. pylori*-associated gastritis, which should be further studied in future investigations.

## Data Availability

The data presented in the study are deposited in the GEO repository, accession number GSE230869 and GSE231337.
