# Supplementary material for: Comprehensive analysis of differences in N6-methyladenosine RNA methylomes in Helicobacter pylori infection
Source: Front Cell Dev Biol. 2023 Jun 7;11:1136096. doi: 10.3389/fcell.2023.1136096 (PMC10289286; doi:10.3389/fcell.2023.1136096)

Table S1 The clinical information of patients

|  |  |  |  | *H.pylori* test | |
| --- | --- | --- | --- | --- | --- |
| Patients | Group | Sex | Age | ^14^C | histological |
| Sample1 | control | female | 38 | negative | - |
| Sample2 | control | male | 25 | negative | negative |
| Sample3 | control | female | 58 | negative | negative |
| Sample4 | control | female | 64 | negative | negative |
| Sample5 | *H.pylori* infection | male | 38 | positive | positive |
| Sample6 | *H.pylori* infection | male | 35 | positive | positive |
| Sample7 | *H.pylori* infection | male | 28 | positive | positive |
| Sample8 | *H.pylori* infection | female | 26 | positive | positive |

Table S2. Primer sequences of RNAs for RT-qPCR

| Species | Gene | Forward(5’-3’) | Reverse(5’-3’) |
| --- | --- | --- | --- |
| GES-1 cells | IL-6 | CCCTGAGAAAGGAGACATGTAA | TCTTTTTCAGCCATCTTTGGA |
|  | IL-8 | GCTCTGTGTGAAGGTGCAGT | TTTCTGTGTTGGCGCAGTGT |
|  | VEGF | GCCTTGCCTTGCTGCTCTACC | CTTCGTGATGATTCTGCCCTCCTC |
|  | GAPDH | GGTCACCAGGGCTGCTTTA | GGATCTCGCTCCTGGAAGATG |
| Mouse | METTL3 | AGGCTCAATATACCAGTGCTACAG | GATTTCATCCACCCGTTCATAACC |
|  | METTL14 | AATAGCAAAGATGAACAGAGGGAG | CCGTTTTGAGTTTGGAGCAG |
|  | WTAP | AGTTATGGCACGGGATGAGTT | TCCTGCTGTTGCTGCTTTAGT |
|  | FTO | TGTCCTCAATGACTCAGACGATGG | AGAACTGCCTCAGCCACTCAA |
|  | ALKBH5 | GCGGTCATCATTCTCAGGAAGA | CTGACAGGCGATCTGAAGCATA |
|  | BOLA | ACACGACTCTGAACCGTTGC | GGCTAAGCACTCGTTCACCA |
|  | PTPN14 | TCGGTAACAAGCTTCGCTCG | GCAATGACCAGGACAGAGACA |
|  | ADAMTS1 | TTGAATGGTGTGAGTGGCGA | TTGGATTCTGGGGCTTGTCC |
|  | GAPDH | AGGTCGGTGTGAACGGATTTG | GGGGTCGTTGATGGCAACA |

Table S3 Summary of reads quality control

| Sample | Raw_Reads | Valid_Reads | Valid% | Q20% | Q30% | GC% |
| --- | --- | --- | --- | --- | --- | --- |
| Ctrl1_IP | 48408390 | 46943084 | 89.08 | 98.03 | 93.91 | 48.40 |
| Ctrl2_IP | 42763862 | 41679016 | 89.28 | 98.08 | 94.10 | 50.56 |
| Ctrl3_IP | 35342172 | 34424870 | 89.19 | 97.91 | 93.66 | 48.62 |
| Hp1_IP | 44076794 | 43136794 | 89.61 | 97.96 | 93.79 | 48.67 |
| Hp2_IP | 50053204 | 48997986 | 89.98 | 97.94 | 93.75 | 49.06 |
| Hp3_IP | 42832190 | 41817396 | 89.51 | 97.87 | 93.59 | 48.47 |
| Ctrl1_input | 37850500 | 36742242 | 89.70 | 97.99 | 93.84 | 48.89 |
| Ctrl2_input | 42947980 | 42150946 | 91.03 | 97.99 | 93.87 | 51.43 |
| Ctrl3_input | 33781784 | 33145100 | 90.73 | 97.91 | 93.64 | 48.85 |
| Hp1_input | 40644292 | 40225972 | 91.37 | 98.05 | 93.96 | 48.68 |
| Hp2_input | 40156496 | 39695368 | 91.35 | 98.00 | 93.84 | 48.85 |
| Hp3_input | 42708078 | 42275750 | 91.58 | 97.97 | 93.77 | 48.37 |

Table S4 Summary of reads mapping to the reference genome

| Sample | Valid reads | Mapped reads | Unique Mapped reads | Multi Mapped reads |
| --- | --- | --- | --- | --- |
| Ctrl1_IP | 36289148 | 35273794(97.20%) | 26197153(72.19%) | 9076641(25.01%) |
| Ctrl2_IP | 34587348 | 33733490(97.53%) | 23700564(68.52%) | 10032926(29.01%) |
| Ctrl3_IP | 28288682 | 27430684(96.97%) | 21725511(76.80%) | 5705173(20.17%) |
| Hp1_IP | 35983896 | 34937169(97.09%) | 28421737(78.98%) | 6515432(18.11%) |
| Hp2_IP | 42705494 | 41506419(97.19%) | 33685525(78.88%) | 7820894(18.31%) |
| Hp3_IP | 35089468 | 34043816(97.02%) | 27713483(78.98%) | 6330333(18.04%) |
| Ctrl1_input | 32725506 | 31935209(97.59%) | 23580491(72.06%) | 8354718(25.53%) |
| Ctrl2_input | 38683088 | 37763712(97.62%) | 26453815(68.39%) | 11309897(29.24%) |
| Ctrl3_input | 30240970 | 29436044(97.34%) | 22631640(74.84%) | 6804404(22.50%) |
| Hp1_input | 36611128 | 35773669(97.71%) | 28132070(76.84%) | 7641599(20.87%) |
| Hp2_input | 36988728 | 36129478(97.68%) | 28449771(76.91%) | 7679707(20.76%) |
| Hp3_input | 38462212 | 37547454(97.62%) | 29472757(76.63%) | 8074697(20.99%) |

| Gene ID | Peak Start | Peak End | Chromosome | Log2 (Fold Change) | Annotation |
| --- | --- | --- | --- | --- | --- |
| ENSG00000183153 | 40362590 | 40362769 | chr17 | 6.49 | 3' UTR |
| ENSG00000228251 | 112589153 | 112614233 | chr2 | 5.81 | 3' UTR |
| ENSG00000196834 | 130459605 | 130459784 | chr2 | 5.78 | 3' UTR |
| ENSG00000115267 | 162310568 | 162310897 | chr2 | 5.52 | 3' UTR |
| ENSG00000160471 | 55350176 | 55350596 | chr19 | 5.51 | 3' UTR |
| ENSG00000080007 | 73413634 | 73413962 | chr6 | 5.50 | 3' UTR |
| ENSG00000224281 | 119466046 | 119466315 | X | 5.49 | Exon |
| ENSG00000270049 | 67481404 | 67481614 | chr16 | 5.45 | Exon |
| ENSG00000244479 | 144336783 | 144349106 | chr7 | 5.23 | Exon |
| ENSG00000128578 | 129485611 | 129485881 | chr7 | 5.20 | 3' UTR |
| ENSG00000138029 | 26244917 | 26245156 | chr2 | -6.93 | 5' UTR |
| ENSG00000073969 | 46640090 | 46643106 | chr17 | -5.75 | 3' UTR |
| ENSG00000181625 | 29457636 | 29458189 | chr16 | -4.54 | 3' UTR |
| ENSG00000213648 | 29457634 | 29461466 | chr16 | -4.49 | 5' UTR |
| ENSG00000168234 | 24133184 | 24133274 | chr18 | -4.29 | 3' UTR |
| ENSG00000197978 | 82436284 | 82436374 | chr15 | -4.22 | Exon |
| ENSG00000279457 | 188902 | 195381 | chr1 | -4.19 | Exon |
| ENSG00000221533 | 154887360 | 154887458 | X | -4.16 | Exon |
| ENSG00000118997 | 196068358 | 196068717 | chr2 | -3.91 | 5' UTR |
| ENSG00000114544 | 126085862 | 126085951 | chr3 | -3.81 | Exon |

Table S5 The top 20 differently expresses m6A peaks between *H. pylori* and controls based on Log2 (Fold Change)

Figure S1. *H. pylori* colonized the stomach in C57BL/6mice.


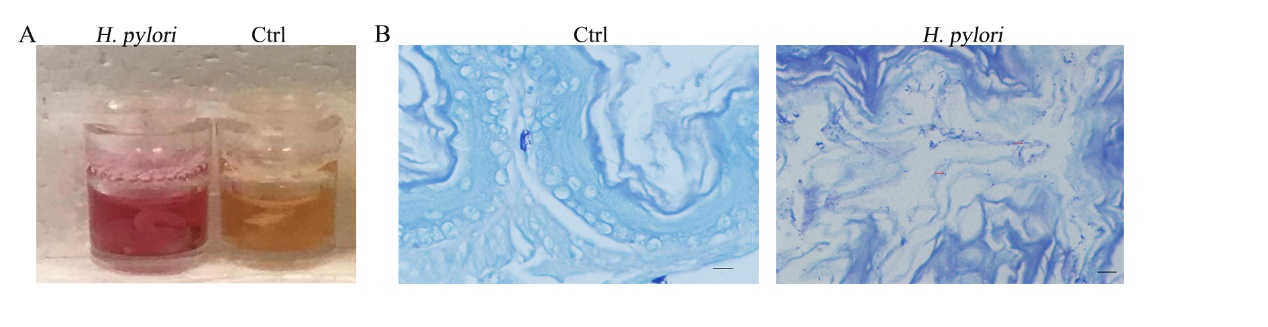


Mice were tested for *H. pylori* infection using RUT and Giemsa staining 7 days after the last intragastric gavage. (A) RUT showed that the bacteria were reactive positively for urease. (B) Geimsa staining showed the characteristic spiral-shaped Helicobacter pylori organisms (red arrows) were present in mouse stomach (scale bars: 20 µm).

Figure S2. Refer to the genome to compare the regional distribution


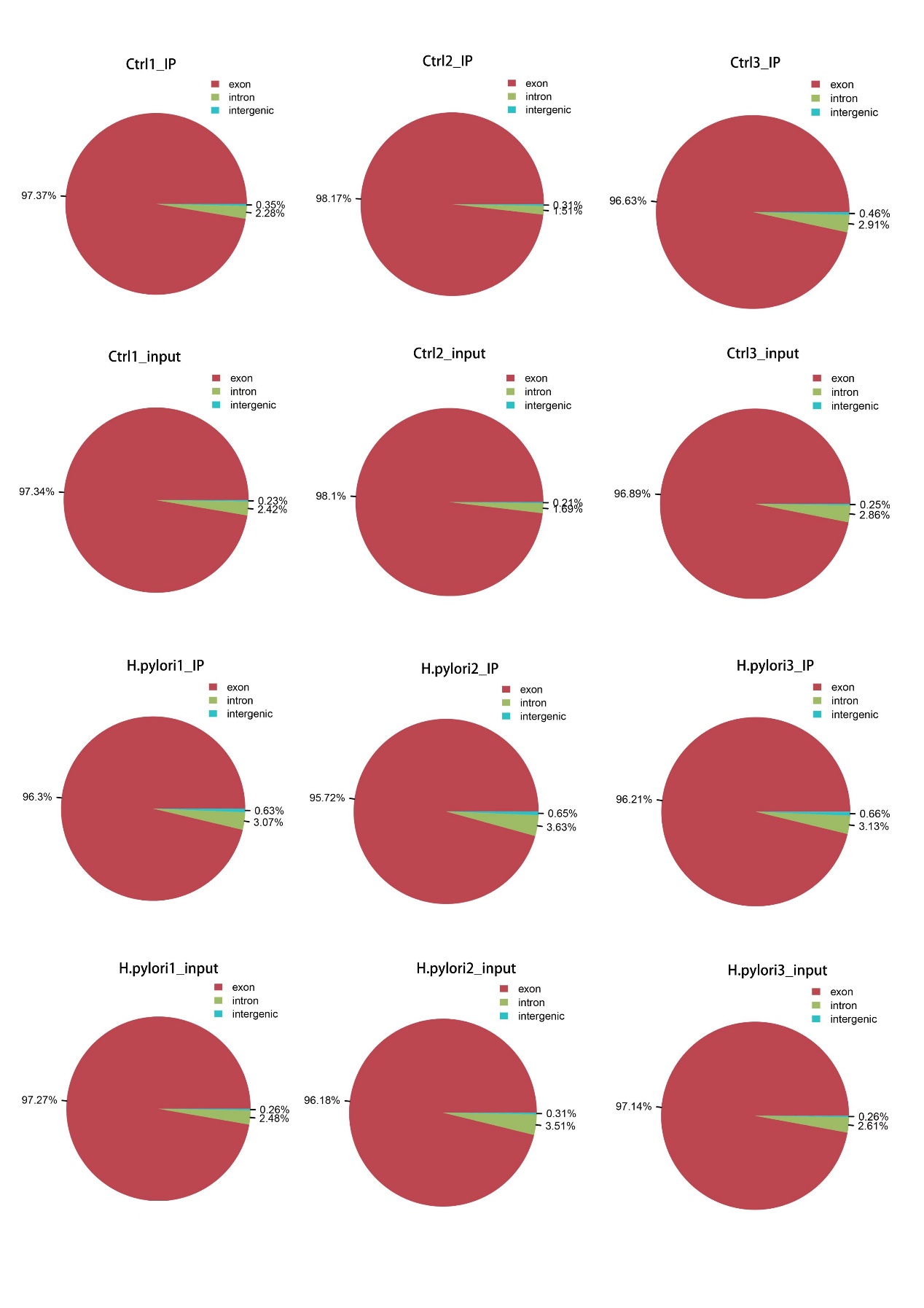

Supplement: Supplementary file 1 [file Table1.DOCX]
